# RAASi Therapy Attenuates the Association between 24-h Urinary Potassium Excretion and Dietary Potassium Intake in CKD Patients

**DOI:** 10.3390/nu15112454

**Published:** 2023-05-24

**Authors:** Domenico Giannese, Claudia D’Alessandro, Nicola Pellegrino, Vincenzo Panichi, Adamasco Cupisti

**Affiliations:** Department of Clinical and Experimental Medicine, University of Pisa, 56126 Pisa, Italy

**Keywords:** diet, RAAS inhibitors, CKD, potassium, urine potassium

## Abstract

The aim of this study was to evaluate urinary potassium (K) excretion as a reliable marker of dietary K intake, in a cohort of CKD patients with or without Renin-Angiotensin-Aldosterone System (RAAS) inhibitor therapy. One hundred and thirty-eight consecutive out-patients (51 f and 87 m) aged 60 ± 13 years and affected by CKD stage 3–4, who were metabolically and nutritionally stable, entered the study between November 2021 and October 2022. No difference was observed between patients with (*n* = 85) or without (*n* = 53) RAAS inhibitor therapy, regarding dietary intakes, blood biochemistry, and 24-h urine excretion parameters. Considering all patients, urinary K showed a weak relationship with eGFR (r = 0.243, *p* < 0.01), and with dietary K intake (r = 0.184, *p* < 0.05). Serum K was not associated with dietary K intake, but an inverse relationship was observed with eGFR (r = −0.269, *p* < 0.01). When patients were examined depending on whether they were receiving RAAS inhibitor therapy, the weak inverse relationship between serum K and eGFR was maintained in both groups. Conversely, urinary K excretion remained positively associated with dietary K intake only in the no RAAS inhibitor group. In conclusion, 24-h urine K excretion may be used as a surrogate of K intake, but RAAS inhibitor therapy reduces the association between 24-h urine K excretion and dietary K intake in CKD patients.

## 1. Introduction

In the general population, a high dietary intake of potassium (K) seems to protect the cardiovascular system, and the World Health Organization and the American Heart Association recommend a daily K intake of 90–120 mEq as safe and effective for cardiovascular protection [1].

Evidence exists to support the strong association between K consumption and arterial blood pressure [2]. The potassium-dependent reduction in blood pressure is mainly attributed to the natriuretic effect due to a decreased sodium reabsorption in the proximal and distal renal tubule. In addition, endothelium-mediated and neuroendocrine vasodilatation induced by potassium is also advocated [3]. Moreover, high potassium intake reduces the risk of having a stroke. In a meta-analysis that included 22 randomized control trials, Aburto et al. reported the favorable impact of potassium intake on arterial blood pressure and a statistically significant reduction in the risk of having a stroke [4]. The cardiovascular protective effects of potassium intake are also known to have a role in major cardiovascular events: a low potassium to sodium intake ratio increases the risk of cardiovascular and ischemic heart disease [5]. Chang et al. confirmed the protective role of high potassium intake reporting that the replacement of sodium chloride with potassium chloride reduced cardiovascular events and related mortality [6,7].

The role of potassium intake in chronic kidney disease (CKD) patients has usually been considered in conditions at risk of hyperkalemia, such as CKD stage IV and V, where a restriction of potassium dietary intake is often required. Furthermore, low potassium intake, when it is not derived from vegetable sources, has been shown to be a strong predictor of mortality regardless of the level of kidney function [8]. Conversely, high dietary potassium has a protective effect on early CKD progression [9,10,11] and on the onset of albuminuria during a long follow-up period [12]. Instead, contrasting data exist in patients with CKD stage III-V who are not on dialysis. Some studies using potassium urinary excretion as a surrogate of dietary potassium intake reported an unfavorable effect of low potassium intake on CKD progression risk [13,14].

Patients with acute or chronic reduction in kidney function are at a higher risk of hyperkalemia. This condition may predispose them to arrhythmias and sudden death; it is a well-known concern for both patients and nephrologists. It is conceivable in an emergency setting or in the case of acute kidney injury, but it has been demonstrated that chronic hyperkalemia is also associated with an increased risk of death with a positive relationship with K serum levels. This is a major problem that arises during the daily clinical management of CKD patients [15], i.e., the control of K serum levels together with a high dietary intake of K.

Several factors other than dietary intake may affect the internal and external balance of potassium which in turn induces changes in serum potassium levels. Accordingly, several investigations demonstrated that dietary potassium was not associated with serum potassium or hyperkalemia in either CKD or in end-stage kidney disease [16].

Potassium in food is almost completely absorbed in the first tract of the small intestine, however, the energy-driven potassium excretion by the colon leads to high K concentration in the fecal water [17].

To maintain a neutral K external balance [18], kidneys regulate K excretion which accounts for about 90% of the K introduced by diet, whereas the remaining 10% is eliminated in the feces [17]. This is why urine K excretion is considered a surrogate of dietary intake. However, this is the case when fecal excretion is stable, and the internal K balance is neutral. These two issues may be reasonably accepted in healthy subjects, whereas they may be significant confounding factors in CKD patients.

Furthermore, CKD patients are often advised to limit K intake to reduce the risk of hyperkalemia [18,19]. In patients with severe reduction in kidney function, when glomerular filtration rate (GFR) drops lower than 20 mL/min, K excretion per single nephron, namely the excretion fraction for K, dramatically increases as well as fecal excretion that reaches up to 30% of ingested K [20].

Hence, the regulation of urinary potassium excretion is the main mechanism that allows a neutral K external balance [21]. This role may be impaired by a substantial reduction in kidney function and/or by drugs that can influence tubular K handling, namely Renin Angiotensin Aldosterone System (RAAS) inhibitors. RAAS blockers affect potassium handling in the distal and collecting tubule. Thus, urine excretion might be influenced by this class of medications, jeopardizing the neutrality of external potassium balance. The question arises whether urine K excretion may still be considered a surrogate for dietary K intake even in CKD patients treated with RAAS inhibitors.

The aim of this study was to evaluate the 24-h urine K excretion as a reliable marker of dietary K intake, in a cohort of CKD stage 3–4 patients with or without RAAS inhibitor therapy.

## 2. Subjects and Methods

One hundred and thirty-eight out-patients (51 f, 87 m) aged 60 ± 13 years affected by CKD stage 3–4, metabolically and nutritionally stable, entered the study during the period of November 2021–October 2022.

Patients with severe cardiac failure (stage IV NYHA), respiratory or liver insufficiency, cancer, dementia, psychiatric or neurologic diseases, inflammatory systemic diseases, or patients who did not give their consent to the study were excluded. All the patients who took part in the study underwent standard biochemistry including determinations on 24-h urine collection and dietary assessment.

Patients were divided into two groups according to whether they were receiving RAAS inhibitor therapy, which included angiotensin-converting enzyme (ACE) inhibitors, angiotensin 2 receptor blockers, aldosterone receptor antagonists, or renin inhibitors.

The study protocol was approved by the local Ethics Committee of the University Hospital of Pisa.

### 2.1. Biochemistry

Biochemistry included serum potassium, creatinine, blood urea nitrogen (BUN), phosphorus, calcium, albumin, bicarbonate, parathyroid hormone (PTH), and blood cell count. All the tests were performed with standard methods routinely used in our laboratory. In particular, the quantitative determination of K in plasma and urine was performed using ion-selective electrodes (Cobas c-701 analyzer, Roche, Penzberg, Germany).

The glomerular filtration rate was estimated (eGFR) using the CKD-EPI formula [22]. Urinary potassium, sodium, phosphate, and urea were measured on 24-h urine samples. Patients had been carefully instructed to perform a correct 24-h urine collection, with the help of a cartoon, a visual tool that simplifies the explanation: in summary, they were instructed to discard the first morning void and collect all the urine during the following 24 h. Urine was collected in 2.5 L plastic containers without adding any preservatives. Two non-consecutive collections of 24-h urine were performed, and the value reported for each patient represented the average of the two determinations. Urine creatinine was assumed as a marker for the adequacy of the urine collection procedure: namely 24-h collection with urine creatinine lower than 10 mg/kg/d or higher than 30 mg/kg/d were excluded from the analysis. The protein catabolic rate was calculated with the Maroni–Mitch formula to estimate dietary protein intake, based on 24-h urinary urea excretion and body weight [23].

### 2.2. Assessment of Dietary Intake

The method used to collect dietary habits consisted of a combination of two tools (a 3-day record + a 3-day recall) and was carried out in two phases. In the first phase, patients meet the dietitian who asks them to register a 3-day record, i.e., write their dietary habits on a prepared form and provides them instructions (oral and written) on how to collect data correctly (indication of the quantities of foods, possible collection of photos of the nutritional facts, and list of ingredients, recipes, and methods of preparation). In the second phase, which is scheduled no more than 5 days from the first meeting, the patient meets the dietitian again who, based on the information registered by patients in the form, carries out an interview repeated for three days (3-day recall starting from the 3-day record). During the interview, the dietitian registers a list of foods that are usually consumed, then provides a more detailed description of the food and beverages consumed, using food models or photos from a food atlas [24] and compares this information with data obtained from the 3-day data collected by patients at home.

Hence, food composition data were obtained using the latest food composition tables of the European Institute of Oncology [25]. The mean of the 3-day dietary habits collection was assumed as the intake of each patient.

### 2.3. Statistical Analysis

Results are reported as mean ± standard deviation or median and inter-quartile range when appropriate. Normal distribution was assessed with the Kolmogorv-Smirnov test. Statistical analysis was performed using the *t*-test for the unpaired sample (two-tailed), or using the Mann-Whitney test, when appropriate. To compare quantitative variables with continuous data Pearson’s correlation analysis was performed, whereas the non-parametric Spearman’s rank correlation test, was used when appropriate. Differences were considered statistically significant when *p* < 0.05. All analyses were carried out by SPSS v.26 technology (IBM, Armonk, NY, USA).

## 3. Results

Table 1 shows the clinical features of the patients studied by use of RAAS inhibitor drugs. According to the selection criteria, all the patients were metabolically stable and free from disabilities or overt protein and energy wasting. Thirty-one (22.4%) out of the 138 patients had diabetes. Eighty-five patients out of the 135 patients studied were taking RAAS inhibitors (61.6%). The 22.5% of patients in the no RAAS inhibitor group and the 17.6% of patients in the RAAS inhibitor group were taking furosemide.

No differences were observed between patients with or without RAAS inhibitor therapy, regarding body weight, kidney function, electrolytes and bicarbonate serum levels, hemoglobin, and all the other studied urinary parameters as reported in Table 1.

Similarly, daily dietary intake resulting from dietary habits assessment and derived food composition analysis were also similar between patients with or without RAAS inhibitor therapy: no statistical difference between the two groups was detected (Table 2).

Considering all the patients studied, urinary K excretion rate showed a weak relationship with only eGFR (ρ = 0.208, *p* = 0.005) and dietary K intake (ρ = 0.184, *p* = 0.0326). Serum K levels were not associated with dietary K intake, whereas an inverse relationship was observed with eGFR (ρ = −0.269, *p* = 0.0016).

As expected, dietary K intake was closely related to fiber intake (ρ = 0.488, *p* < 0.0001).

**Figure 1:**
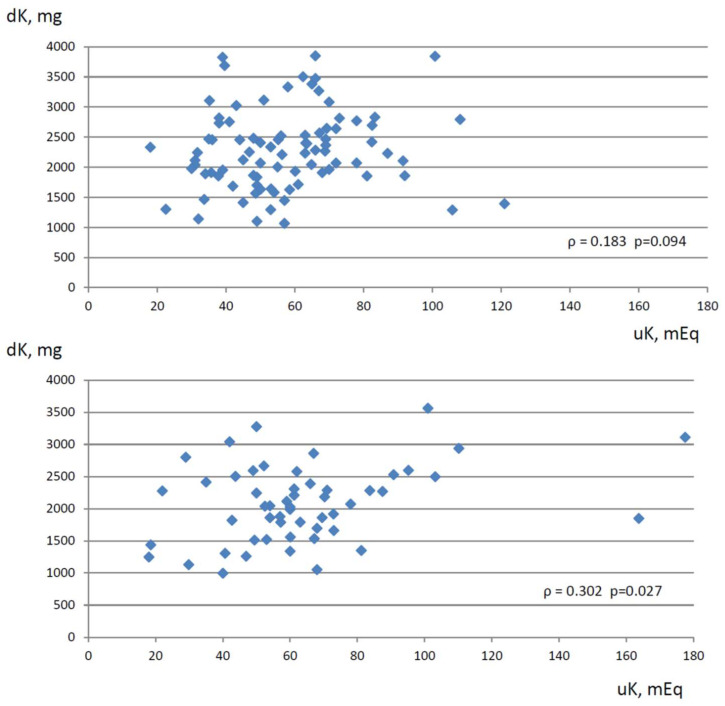
Relationship between dietary K intake (expressed as mg/d, Y-axis) estimated by 3-day dietary habits collection and 24-h urine K excretion (expressed as mEq/d, X-axis) in CKD patients on RAAS inhibitor therapy (upper graph) and in CKD patients with no RAAS inhibitor therapy (lower graph). ρ: Spearman’s rank correlation coefficient.

When patients were examined separately, depending on whether they were receiving RAAS inhibitor therapy, the weak relationship between serum K levels and eGFR values was maintained in both groups (Table 3). Instead, urinary K excretion resulted statistically associated with dietary K intake only in the group not taking RAAS inhibitor medications, whereas the positive relationship between urinary K excretion and dietary K intake was missing in the RAAS inhibitors group (Figure 1). Similarly, urinary K excretion was positively related to eGFR only in the patients not receiving RAAS inhibitor therapy, whereas, in patients on RAAS inhibitor therapy, the relationship was absent.

## 4. Discussion

The main findings of our study are that urine K excretion may still be considered a weak surrogate of dietary K intake in patients with a moderate to severe reduction in kidney function and that the weak relationship between dietary K intake and urine K excretion is maintained if patients do not take RAAS inhibitor medications. In other words, it seems that RAAS inhibitor therapy blunted the relationship between the amount of urinary K excretion and dietary K intake.

Since external K balance is regulated by the kidney, urine K excretion is considered a surrogate for the estimation of dietary input based on the assumption that external balance is neutral. This may also be true when Ks internal balance is neutral, but unfortunately, we do not have a tool to assess the internal balance of K in clinical practice. It is noteworthy that internal balance is affected by several metabolic and hormonal factors, and the potential changes may be relevant due to the high K concentration in the intracellular space.

Several studies used 24-h urinary K excretion to estimate dietary K intake. This is well accepted for individuals with preserved kidney function, and evidence exists in the literature. For instance, a two-week controlled-feeding study including postmenopausal women demonstrated that 24-h urine collection performs better than estimated 24-h excretion from spot urine as a biomarker for average daily dietary sodium and K intake [26].

In a cohort of healthy adolescents, K intakes determined by the 24-h dietary recall were weakly correlated with 24-h urine K excretion measurements (r = 0.229) [27].

A significant positive relationship was found between 24-h urinary K and dietary healthy habits by using a 2-day dietary recall in a cohort of healthy individuals. Self-reported K intake and 24-h urinary K excretion were positively, but weakly, related (Spearman’s correlation coefficient = 0.233; *p* < 0.001) [28].

In adults with normal renal function, 24-h urine K excretion measurements were found to be statistically (although weakly) correlated with dietary intake derived from food frequency questionnaires (r = 0.260; *p* < 0.001) [29]. Urinary K is significantly related to BMI, blood pressure, and heart rate. The authors concluded that urinary K provides a summary measure of diet quality and may be clinically useful to detect poor dietary habits and monitor response to overall dietary interventions.

In a small cohort of healthy people studied in a metabolic suite, dietary K intake was found to be highly correlated with urinary K excretion [30]. Similarly, to healthy individuals, the estimation of 24-h excretion of sodium and K using sodium and K dosage on spot urine is a poor indicator of true sodium and K intake; in the CKD population also [31].

A randomized trial including stage 3 CKD patients reported the effects of different dietary K intakes on arterial blood pressure and K serum levels. Regarding urine excretion, the lower-K diet group (40 mmol/d) had a K excretion rate of 39.9 mmol/24-h whereas the higher-K diet group (100 mmol/d) had a K excretion of 81.4 mmol/24-h. These data demonstrated different K excretions according to K dietary intake in patients with a moderate reduction in kidney function [32].

Although reduced kidney function may impair the capacity to handle a dietary K load, urine K excretion is used to estimate dietary K intake also in the CKD population [18,33,34]. However, the use of RAAS inhibitors in CKD patients is highly prevalent and it is well known that there is a risk of hyperkalemia due to impaired renal excretion. Therefore, it begs the question of whether urine K excretion may still be considered a surrogate for dietary K intake also in patients with CKD on RAAS inhibitor therapy.

Our study suggests that this is not true in patients affected by CKD stages 3–4 on RAAS inhibitor treatment. This finding is a novelty because none of the studies considering 24-h urine K excretion as a surrogate of dietary K intake included and recognized this bias. This may be of concern for further research that would estimate K intake using K renal excretion in CKD patients on RAAS inhibitor therapy.

Moreover, RAAS inhibitors impair K handling in the kidney distal tubule, thus limiting the kidney’s capacity to excrete a K load.

Our study also shows some other confirmatory findings. K serum levels were related to residual kidney function, as expected: the risk of hyperkalemia increases as a negative function of eGFR [35]. A direct linear relationship was clearly reported by Inker et al. in a large CKD population [36]. Moreover, both acute kidney injury and chronic renal insufficiency are major determinants for the onset of hyperkalemia [37].

The level of dietary K was also closely associated with fiber content in the diet, namely to plant-origin foods, which are the most relevant sources of K. It is noteworthy that plant or animal food with the same K content may differ in how they contribute to the increase in serum K levels up to hyperkalemia [38]. Namely, the lower net fixed acid production induced by plant foods with respect to animal ones counteract metabolic acidosis limiting the extracellular K shift and consequently, the risk of hyperkalemia.

In addition, plant-derived foods are rich in fibers that increase intestinal motility so reducing the risk of constipation, which remains a major risk factor for hyperkalemia even in a hemodialysis patient. Finally, clues exist that K of plant origin may be less accessible and possibly less bioavailable [39]. In summary, different sources of dietary K, namely plant-based or animal-based foods, may exert opposite effects on K serum levels in CKD [40,41].

Therefore, it is not surprising that no relationship between the amount of dietary K intake and serum K was detected in our studied CKD population. This is reported in several studies in CKD settings even in its severe stages, including dialysis [42]. These data further support the most recent guidelines about nutrition in CKD patients that recommend a reduction in K intake only in the case of hyperkalemia and not based on residual kidney function per se [41].

Regarding the renal regulation of K homeostasis, aldosterone represents the main factor influencing K tubular excretion. It is well known that aldosterone stimulates Na reabsorption in the distal tubule and cortical collecting duct, both coupled with the excretion of K. However, to date, reports examining the relationship between RAAS inhibitor use and urinary K+ excretion in advanced CKD patients are limited. In the setting of a substantial reduction in eGFR, the RAAS has a relevant role in renal tubule K handling and RAAS inhibition has been easily associated with the increment of K serum levels in CKD patients. The risk of raising K serum levels is associated with a decrease in urinary K excretion capacity due to the reduced glomerular filtration rate and with a K redistribution from intracellular to extracellular fluid caused by the impaired renal acid excretion capacity. Previous studies reported no significant changes in urinary K excretion, and fractional excretion of K before and after initiation of RAAS blockers, namely both ACE inhibitors and ARBs [43,44]. In a recent cross-sectional retrospective study including 691 patients affected by CKD, Ueda et al. confirmed that RAAS blocker therapy was associated with hyperkalemia and that there was no difference in urinary K excretion among the groups with or without RAAS inhibitors [45]. More likely, the authors attributed the increased K serum levels to changes in K redistribution from intracellular to extracellular fluid induced by the metabolic acidosis occurring in patients with CKD stages G3b and G4 [45]. Thus, changes in internal K balance rather than in external K balance may explain the risk of hyperkalemia.

It is noteworthy that in our series, 61.6% of CKD outpatients were on RAAS blocker therapy. The prevalence of RAAS blockers in our study is quite similar to that reported in the Chronic Kidney Disease Outcomes and Practice Patterns Study (CKDopps), including 5870 patients from different countries, where RAAS blocker prescriptions ranged from 80% in Germany to 52% in the United States [46]. The use of RAAS inhibitors does not seem to be so high, possibly due to under-prescription or to the occurrence of episodes of hyperkalemia. Moreover, our data are well in keeping with a European survey where a RAAS inhibitor prescription was reported in 60.5% of the patients who had one episode of hyperkalemia as assessed by K serum level >5 mEq/L [47].

Finally, dietary K intake has been evaluated by using self-reported dietary intakes. This cannot be considered the gold standard, but it is the best we have in a clinical practice setting. To make an estimation of nutrient intake in the best way possible, the procedure of the 3-day dietary habits collection used in this study included due phases. As reported in the methods section, in the first phase patients meet the dietitian who asks them to register their dietary habits on a prepared form and gives instructions, delivered orally or in written form, on how to achieve a correct collection of data, namely an indication of the amount of food, possible collection of photos of the nutritional facts, and list of ingredients, recipes, and methods of preparation. In the second phase, which is scheduled no more than 5 days from the first meeting, the patient meets the dietitian again who, based on the information that patients have registered in the form, carries out an interview that simulates the 24-h dietary recall multipass method [48], but is repeated for three days. During the interview, the dietitian registers a list of food that is usually consumed, then provides a more detailed description of food and beverages consumed using food models, or photos from a food atlas, and compares this information with data obtained from the 3-day data collected by patients at home. The strength of this method is that it provides quantitative estimates of individual food consumption and nutrient intake, gives information on food sources and preparation methods, and offers a higher degree of accuracy in assessing food and nutrient intake relative to a food frequency questionnaire. It requires a skilled dietitian with a high level of training to conduct a good interview and minimize errors in data collection. Overall, this method reduces memory bias such as forgetting certain food or information such as the amount or type of dressing, which is one of the most common things that are forgotten, or the cooking method or the type of fruits or vegetables. This is particularly important in this study focusing on K dietary intake.

## 5. Conclusions

Although urine K excretion is still considered an indicator of K intake in subjects with or without reduction in kidney function, our study suggests that pharmacological therapy including RAAS inhibitors reduces the association between urinary K excretion and dietary K intake, at least in patients with stage 3–4 CKD. Our conclusion needs to be confirmed by studies assessing dietary K intake with more reliable methods (more reliable than dietary habit collection) performed with the common methods used in daily clinical practice. However, we believe that caution should be used when dietary K intake is estimated by urine K daily excretion in the CKD population on RAAS blockers.

## Figures and Tables

**Table 1:** Clinical features of the patients studied, by Renin Angiotensin Aldosterone System inhibitors (RAASi) therapy.

	RAASi	RAASi	
	NO	YES	*p*
Age, years	59.4 ± 14.0	61.1 ± 13.3	0.508
Weight, kg	77.8 ± 13.8	77.0 ± 15.5	0.743
BMI, kg/m^2^	28.5 (25.7–30.2)	25.4 (24.0–28.9)	0.022
BUN, mg/dL	28.7 (24.7–39.0)	34.3 (25.7–45.8)	0.056
sCreatinine, mg/dL	1.60 (1.36–2.27)	1.83 (1.54–2.55)	0.058
eGFR, mL/min/1.73 m^2^	42.0 (28.5–49.6)	32.8 (24.0–43.8)	0.052
sSodium, mEq/L	141 (139–143)	142 (140–143)	0.072
sPotassium, mEq/L	4.6 (4.2–4.8)	4.7 (4.3–5.0)	0.103
sCalcium, mg/dL	9.6 (9.3–10.1)	9.5 (9.2–9.9)	0.202
sPhosphate, mg/dL	3.4 ± 0.7	3.3 ± 0.6	0.626
sBicarbonate, mEq/L	25.0 (23.9–28.3)	24.0 (22.1–26.0)	0.059
sAlbumin, g/dL	4.1 (3.88–4.35)	4.2 (2.75–4.39)	0.825
Hemoglobin, g/dL	13.3 ± 1.6	13.3 ± 1.8	0.950
PTH, pg/mL	47.0 (34.0–62.4)	69.8 (45.1–114)	0.011
uCreatinine, mg/24-h	1186 (933–1431)	1200 (1002–1538)	0.481
uUrea, g/24-h	19.2 (15.1–24.0)	18.4 (15.0–23.0)	0.718
uSodium, mEq/24-h	149 (105–200)	134 (101–170)	0.147
uPotassium, mEq/24-h	60.0 (49.2–72.0)	56.0 42.5–68.9)	0.228

Data reported as mean ± SD or median and inter-quartile range (Q1–Q3) when appropriate.

**Table 2:** Dietary intake features of the patients studied, by Renin Angiotensin Aldosterone System inhibitors (RAASi) therapy.

	RAASi	RAASi	
	NO	YES	*p*
Energy, Kcal	1833 (1402–2156)	1833 (1464–2063)	0.903
Protein, g	69.4 ± 21.6	62.7 ± 17.4	0.084
Lipids, g	60.5 (48.5–75.0)	63.8 (48.2–76.8)	0.604
Carbohydrates, g	248 (170–285)	231 (190–295)	0.942
Sodium, mg	948 (632–1691)	1109 (719–1658)	0.440
Potassium, mg	2077 ± 592	2284 ± 656	0.063
Calcium, mg	521 ± 230	502 ± 225	0.634
Phosphorus, mg	970 ± 280	958 ± 362	0.827
Iron, mg	8.0 (6.25–10.6)	8.7 (6.55–10.6)	0.546
Fibers, g	16.0 (10.4–20.2)	16.8 (12.3–19.3)	0.482

Data reported as mean ± SD or median and inter-quartile range (Q1–Q3) when appropriate.

**Table 3:** Spearman’s rank correlation analysis (expressed as ρ values) among daily urinary K excretion (uK), K serum levels (sK), dietary K intake (dK), and estimated glomerular filtration rate (eGFR) in patients with (bold) or without (italics) Renin Angiotensin Aldosterone System inhibitors (RAASi).

	RAASi Group				
		uK	sK	dK	eGFR
*No RAASi Group*	*uK*	**xxxxxx**	**0.042**	**0.183**	**0.154**
	*sK*	*0.063*	**xxxxxx**	**−** **0.045**	**−** **0.232 ^**
	*dK*	0.302 *	−*0.072*	**xxxxxx**	**0.056**
	*eGFR*	0.343 ***	−0.322 **	*0.216*	**xxxxxx**

No RAASi group: * *p* = 0.027; ** *p* = 0.015; *** *p* = 0.012. RAASi group: ^ *p* = 0.034.

## Data Availability

The data presented in this study are available on request from the corresponding author.
